# Multitasking on the run

**DOI:** 10.7554/eLife.00641

**Published:** 2013-03-19

**Authors:** Mary E Hatten, Stephen G Lisberger

**Affiliations:** 1 **Mary E Hatten** is at the Laboratory of Developmental Neurobiology, The Rockefeller University, New York, United Stateshatten@rockefeller.edu; 2 **Stephen G Lisberger** is at the Department of Neurobiology and the Howard Hughes Medical Institute, Duke University School of Medicine, Durham, United Stateslisberger@neuro.duke.edu

**Keywords:** cerebellum, corollary discharge, proprioception, Mouse

## Abstract

Researchers combine genetics and imaging to reveal that individual granule cells in the cerebellum integrate sensory and motor information.

**Related research article** Huang C-C, Sugino K, Shima Y, Guo C, Bai S, Mensh BD, Nelson SB, Hantman AW. 2013. Convergence of pontine and proprioceptive streams onto multimodal cerebellar granule cells. *eLife* **2**:e00400. doi: 10.7554/eLife.00400

**Image** 3D reconstruction of mossy fibers (green and red) terminating at a granule cell

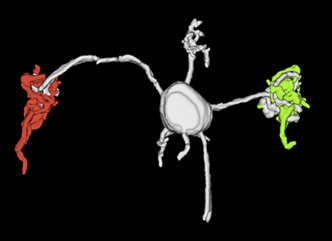

The learning abilities of the cerebellum, also known as the ‘little cortex’, enable us to learn and execute complex motor movements without even thinking about them. Thus, our abilities to play a musical instrument, for example, or even just to reach for a cup of coffee without looking up from our computer, all depend on the cerebellum. One of the hallmarks of the cerebellum, which is hidden under the much larger cerebral cortex, is its simple, stereotyped architecture and well-understood circuitry. This simplicity means that analysis of the structure and function of the cerebellum offers neuroscientists the opportunity to understand the basic principles of cortical function.

For many years, it has been known that the cerebellum relies on two fundamental principles of operation. First, it is able to compare sensory signals (which arise in sensory receptors and report what has happened in the outside world) and motor signals (which are corollaries of the motor commands sent to the spinal cord). Second, ‘plasticity’ in the cerebellum mediates the learning of motor skills. Here, plasticity refers to changes in the strength of the synapses that allow one neuron to communicate with another, or to changes in the number of action potentials emitted by a neuron when it receives a given set of synaptic inputs.

Writing in *eLife,* Adam Hantman and colleagues—including Cheng-Chiu Huang as first author—provide direct evidence that link these two principles. They show that sensory and motor inputs to the cerebellum converge at the earliest stage of processing, on the cerebellar granule neuron (Huang et al., 2013). Their findings have important implications for how the cerebellum does its job, including how and where synaptic plasticity causes learning of motor skills.

The cerebellar cortex consists of three layers—an innermost layer of granule neurons, a central layer of Purkinje cells, and an outer molecular layer—and it receives two major sources of input: mossy fibers and climbing fibers. Mossy fibers originate in multiple regions, relaying motor and sensory inputs to synapses on the dendrites of granule neurons (Palay and Chan-Palay, 1974). The axons of the granule neurons ascend through the Purkinje neuron layer into the molecular layer, where they bifurcate into ‘parallel fibers’ and form synapses with the dendrites of Purkinje neurons (Figure 1). Tens, or maybe hundreds, of thousands of parallel fibers can form synapses with a single Purkinje cell.

**Figure 1.**
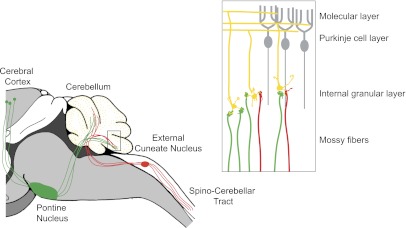
The cerebellum integrates motor and sensory signals, which allows us to learn and perfect complex motor skills. The signals are relayed to the cerebellum by mossy fibers: copies of motor commands sent by the cerebral cortex are relayed via the pontine nucleus (green), while sensory signals are relayed from the spinal cord via the external cuneate nucleus (red). Inset: Expanded view of the cerebellum showing mossy fibers (red and green) that have formed synapses with the dendrites of granule neurons (yellow). More than 40% of the granule cells studied by Huang et al. received inputs from both sensory (red) and motor (green) mossy fibers. The axons of the granule neurons (yellow) ascend into the molecular layer of the cerebellum, where they bifurcate to form parallel fibers: large numbers of parallel fibers can form synapses with a single Purkinje cell (gray). The climbing fibers are not shown.

FIGURE: MARY E HATTEN/JIM DUFFY

Climbing fibers, on the other hand, originate from the inferior olivary nucleus in the brainstem. Moreover, each Purkinje cell receives many synaptic contacts from only a single climbing fiber. This unusual anatomical feature has driven much of the research into the function of climbing fibers.

Classical models of cerebellar function—first proposed independently by David Marr in 1969, James Albus in 1971 and Masao Ito in 1972 (Marr, 1969; Albus, 1971; Ito, 1972)—hold that the electrical activity of mossy fibers acts on a millisecond-by-millisecond basis to create a pattern of Purkinje cell action potentials that guides accurate movement, while climbing fibers produce action potentials on a longer time scale (about once per second) to guide plasticity in the cerebellum. Plasticity adjusts the strength of each parallel fiber input and attempts to create the correct commands for accurate, skilled movement. Indeed, when inputs from parallel fibers and climbing fibers arrive at a given Purkinje neuron at the same time, the strength of the synapse between the parallel fibers and the Purkinje cell is reduced (Ito et al., 1982; Linden et al., 1991). This reduction in the strength of the synapse is called ‘long-term depression'.

Huang, Hantman and colleagues—who are based at the Janelia Farm Research Campus of the Howard Hughes Medical Institute and at Brandeis University—now reveal a previously unknown feature of cerebellar organization that speaks loudly to the possible mechanisms by which the cerebellum can ‘learn' new motor skills. Through deployment of a *tour de force* combination of neuroanatomical, genetic and imaging tools, they show that mossy fibers from sensory and motor relay centers converge onto individual granule neurons. Moreover, they demonstrate that this multimodal convergence occurs across the cerebellum.

Huang et al. injected different reporter virus constructs into two mouse brainstem nuclei that are sources of mossy fiber input to the cerebellum. The construct tagged with a red reporter gene was injected into the external cuneate nucleus, which belongs to the pathway that carries proprioceptive sensory information (that is, information about the position of our limbs in space), and the construct tagged with a green reporter gene was injected into the pontine nucleus, which is part of the pathway that carries copies of motor commands from the cortex to the cerebellum. Confocal imaging of the fluorescently tagged mossy fiber projections revealed considerable convergence of the motor and sensory pathways within the cerebellar granule cell layer.

To determine whether the pathways converged onto individual granule cells, Huang et al. used a second transgenic mouse line, the *TCGO* line, which expresses a yellow reporter in a random subset of granule cells. Using high-resolution confocal imaging, they were able to home in on the sparsely labeled granule cells, and to visualize individual mossy fibers terminating on individual granule neuron dendrites (yellow). Remarkably, up to 40% of the granule cells that they analyzed received inputs from both sensory (green) and motor (red) fibers. This is the first evidence for the convergence of sensory and motor afferents at such an early stage of processing.

The most important implication from the paper of Huang et al. is that sensory–motor integration might occur where the mossy fibers meet the granule neurons, and not where the parallel fibers form synapses with the Purkinje cells in the molecular layer. This raises questions about our current models of cerebellar learning. If learning occurs through changes in the strength of the parallel fiber/Purkinje cell synapse, then the early convergence of motor and sensory signals on granule cells means that the cerebellum may have limited ability to adjust the strengths of sensory and motor signals independently. Or, there might be a critical role for plasticity mechanisms at other sites in the cerebellar circuit (Hansel et al., 2001; Carey and Lisberger, 2002), even at the synapses between mossy fibers and granule cells.

The findings provide elegant support for the unifying model of cerebellar function proposed more than 40 years ago by Marr, Albus and Ito. But while the truth may follow the fundamental principles these pioneering authors espoused, it may be more complex than they imagined. Nevertheless, the application of modern biological technology to known neural circuits, as pioneered by Hantman and co-workers, will boost our understanding of how behavior and learning are organized at the circuitry level.
